# DEIVA: a web application for interactive visual analysis of differential gene expression profiles

**DOI:** 10.1186/s12864-016-3396-5

**Published:** 2017-01-07

**Authors:** Jayson Harshbarger, Anton Kratz, Piero Carninci

**Affiliations:** RIKEN Center for Life Science Technologies, RIKEN Yokohama Institute, 1-7-22 Suehiro-cho, Tsurumi-ku, Yokohama, Kanagawa 230-0045 Japan

**Keywords:** Differential gene expression, RNA-seq, Visualization, Web application, Interactive visual analysis

## Abstract

**Background:**

Differential gene expression (DGE) analysis is a technique to identify statistically significant differences in RNA abundance for genes or arbitrary features between different biological states. The result of a DGE test is typically further analyzed using statistical software, spreadsheets or custom *ad hoc* algorithms. We identified a need for a web-based system to share DGE statistical test results, and locate and identify genes in DGE statistical test results with a very low barrier of entry.

**Results:**

We have developed DEIVA, a free and open source, browser-based single page application (SPA) with a strong emphasis on being user friendly that enables locating and identifying single or multiple genes in an immediate, interactive, and intuitive manner. By design, DEIVA scales with very large numbers of users and datasets.

**Conclusions:**

Compared to existing software, DEIVA offers a unique combination of design decisions that enable inspection and analysis of DGE statistical test results with an emphasis on ease of use.

## Background

RNA-seq [1] and other forms of gene expression profiling such as CAGE [2] are widely used for measuring RNA abundance profiles of various primary cells and cell lines [3]. By comparing the transcript abundance between two states, genes with statistically significant differences in expression levels can be identified [4]. In addition to large-scale, landscape-type analysis of such differentially expressed genes, often leading to long lists of Gene Ontology [5] terms, it is often desired to perform an interactive visual analysis of the results, focusing on comparatively few genes of interest, heavily dependent on the problem domain. While domain experts could perform such an analysis using spreadsheet software, scripting languages or statistical software such as R [6] and Ggobi [7], such an approach often requires implementing custom algorithms. Other systems are embedded within large frameworks [8] which necessitates the user to learn the system first, do not allow the user to upload custom data or are closed source [9].

Experienced bioinformaticians are familiar with existing gene expression profiling tools and, in a fast paced research environment, may perform this analysis often, quickly and routinely using these existing tools. However, sharing the results of DGE analysis with collaborators, including biologists and other researchers that may not be familiar with DE analysis tools, as flat files or static images has limited usability.

Against this background, we saw a need for a software that enables interactive visual analysis of DGE with a strong emphasis on ease of use and ease of deployment, which meets user expectations to a modern web application. To address this need, we have developed DEIVA (Differential Expression Interactive Visual Analysis), a SPA to interactively identify and locate genes in a hexagonal binning (hexbin) density or scatter plot of DGE statistical test results, typically from a DESeq2 [10] or edgeR [11] analysis. In addition to identifying and locating genes, DEIVA allows visitors to download associated data and generated vector images. By providing domain experts (biologists) a means to quickly perform lookups on a differential gene expression test, DEIVA can be of use to bioinformaticians who want to share their results and at the same time make them accessible.

DEIVA can easily be deployed by cloning a Git repository and adding custom datasets, then serving the SPA through any web server. Users can also try out the system through a live instance of DEIVA, including import and visualization of their own datasets [12], containing DGE statistical test results from Kratz 2014 [13]. Standalone desktop applications for various platforms are also available with each release.

## Implementation

### Interface

**Fig. 1:**
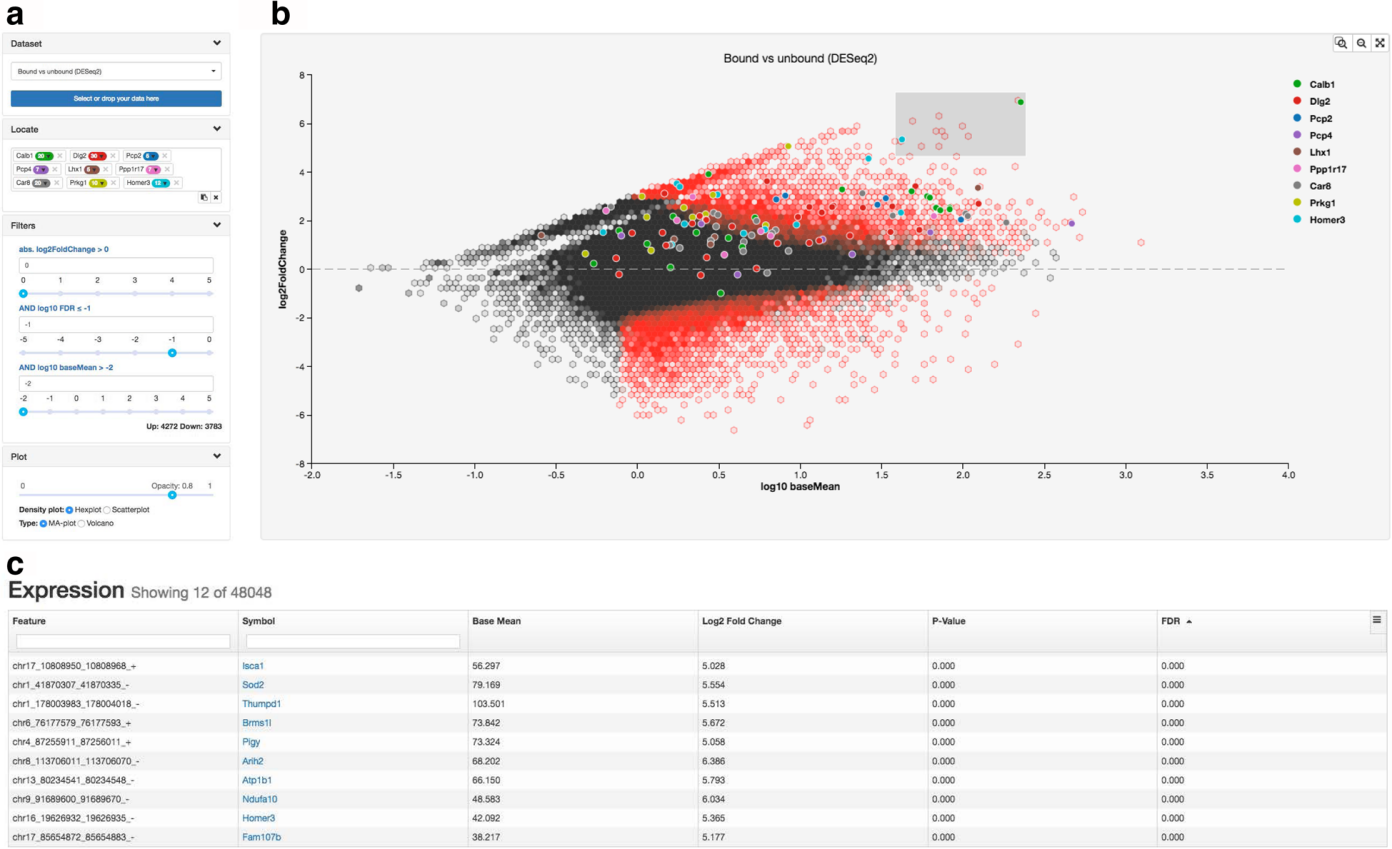
DEIVA interface. **a** Data set selector, symbol locator, and highlight filters. **b** The density plot on a field of log2 FC vs log10 baseMean for a DGE statistical test result. Symbols selected in the symbol locator (shown in (**a**)) are shown as points with matching colors. In this example comparing samples highly enriched for RNA attached to ribosomes of Purkinje neurons (positive fold change) with samples of unspecific RNA in the same brain region (negative fold change). Locating a set of already known markers for Purkinje neurons immediately confirms that the markers are specifically enriched. Hexagonal bins are colored red based on the fraction of features within that region that pass the cut-off filters; currently set at a log10 FDR ≤ −1, at any fold-change. **c** Sortable table of expression values for the region selected in the density plot (shown in (**b**)). Twelve highly overrepresented genes are selected (*grey rectangle*) in the plot and their information is reflected in this table

Figure 1 shows a view of the DEIVA interface. The user may select a pre-loaded DGE statistical test result from the dataset dropdown (Fig. 1a) or drag and drop the user’s own dataset into the visualization area. A density plot of log2 fold change vs. average expression is shown (Fig. 1b). Below the visualization a table of all expression data is displayed (Fig. 1c). Highlighting a region in the visualization limits the features shown in the table to those within that region. Zooming allows easier interaction in crowded regions of the plot.

A user can locate and highlight single or multiple symbols of interest, by typing them into the locate symbol box, selecting them from suggested matches, or by pasting lists of symbols. Such symbols of interest could include genes with expected behavior of fold change or marker genes corresponding to the compared states. In this way the user might see at one glance whether an experiment confirms expectations or needs to be examined in more detail.

To see the effect of more relaxed or stringent criteria for calling a feature differentially expressed, the user can adjust the absolute log2 fold change, False Discovery Rate (FDR) and log10 baseMean cutoff filters using sliders. Features passing these filters will be indicated in red on the plot and the number of up- and down-regulated features will be displayed below the filters.

At any time, the user can download the raw data or the current visualization as publication quality vector graphic in SVG format.

### Input file formats and deployment

DEIVA accepts input files in tab or comma-separated ASCII describing the result of a DGE statistical test. Any algorithm can be used to generate an input file as long as it is possible to export average abundance, log2 fold change, and unique feature names. An optional column “symbol” makes it possible to specify gene symbols independent of the features in which gene expression has been measured (transcription start sites, probes). This accommodates scenarios where one gene may be associated with more than one feature during the DGE test. We anticipate that DEIVA will mostly be used with input generated by DESeq2 [10] and edgeR [11], and DEIVA accepts input files that can be directly written from these R packages. Detailed instructions on preparing files for input are part of the DEIVA documentation.

DEIVA is an open source SPA, not a centralized server application, it is therefore easy to deploy multiple instances each with datasets ready to use directly or to share with collaborators. To deploy a custom instance of DEIVA, a developer may clone the source, add the desired DGE statistical test results, and make the SPA accessible through any web server. DEIVA was developed using Project χ, a modular open-source toolkit for building web and cross platform desktop data visualization applications. Project χ utilizes the AngularJS JavaScript framework, the D3js visualization library [14], and various node.js development tools. The resulting application is compatible with all modern web browsers (we tested with Chrome 51, Firefox 47, MS Edge, and Safari 9) and does not require any specific browser or server dependencies.

## Results and Discussion

We have tested DEIVA with input files ranging from ~50,000 to ~90,000 features with various browsers and operating systems, and find it responsive at these typical file sizes. By default, the visualization will display a hexbin density plot of the differentially expressed values. The user may also switch to a scatter plot view. In general, the density plot has better performance and will result in a more responsive user experience, while the scatter plot displays full detail.

All processing and visualization of the data occurs within the web browser or desktop application. When using a web server, the server is only responsible for sending the SPA code and the data for experiments that are pre-loaded in the given DEIVA instance. If other data is visualized by a user using the interface, the users data is not sent to any server but stays on the client side. The fact that DEIVA is a client-side SPA has several implications:

1. DEIVA can be expected to scale to virtually any number of users and datasets.
2. The fact that data provided by the user is not uploaded to a host server adds to the security of the system, which is important in the context of sensitive data, such as expression profiling of human patient samples.
3. Performance will vary depending on the user's hardware and software combination. We find DEIVA responsive while providing several hundred datasets with over 90,000 features in each dataset. For datasets with considerably more features, server-based systems can be preferable, if the rendering of the visualization is done server-side.

### Comparison of DEIVA with related software

There are other systems with varying scope and functionality available for the exploration and analysis of DGE statistical test results, most notably VisRseq [15], OASIS [9] and DEGUST [16]. We compare DEIVA directly with these systems in a feature matrix (Table 1). The following features are tabulated:

**Table 1:** Summary of competing tools

	Features							Dependencies		
	locate	identify	MA-plot	Volcano plot	web-based	users data	FOSS license	browser	development	server
OASIS	O	O	X	O	O	X	O: LGPLv2	none	NA	NA
VisRseq	X	O	O	O	X	O	-^a^	NA	Java, R	NA
DEGUST	O	O	O	X	O	O^c^	O: GPL v3	none	bash, node.js^b^	none^b^
DEIVA	Δ	O	O	O	Δ	Δ	O: MIT	none	node.js	none^d^

^a^not specified

^b^for analysis back-end DEGUST requires R, Python, node, and Glasgow Haskell Compiler

^c^requires upload to server or custom deployment with analysis backend

^d^DEIVA can run on any HTTP server including WebDav, node HTTP server, python HTTP server, Apache

Δ State of the art

O Feature present

X Feature absent

- **locate**: includes functionality to visually locate the position of the features of at least one symbol.
- **identify**: includes a functionality to identify at least one feature, or a group of features, on the plot.
- **MA-plot**: can render the DGE statistical test result as a MA-plot (i.e. a scatter plot of mean expression vs log fold change).
- **Volcano plot**: can render the DGE statistical test result as a volcano plot (p-value vs fold change).
- **web-based**: yes if the system is a web-based application, no if it is a client side application.
- **users data**: the user can visualize their own datasets.
- **FOSS license**: the system is available under a free and open source software license; the license is listed.
- **dependencies**: listing of browser, development, and server dependencies.

We also examined GenePattern 2.0 [8]. However, the authors were unable to reproduce the volcano plots as described in the documentation [17] using the GenePattern public servers [18].

Another software in this context is iCanPlot [19], a generic library for generating interactive canvas based scatter plots. Canvas based scatter plots generated by iCanPlot provide excellent performance compared to SVG based scatter plots generated using D3 [14] (as implemented in DEIVA), however, iCanPlot generated plots lack some functionality we felt necessary for DEIVA. For example point-by-point inspection of features, high-contrast color highlighting of features, and download of vectorized images. Additionally, iCanPlot has no ability to generate density plots as is the default in DEIVA. It may be beneficial to implement some level of canvas based rendering in DEIVA, however, this should be done without sacrificing DEIVA's current functionality.

## Conclusions

The feature matrix illustrates that none of the other comparable systems available has the combination of design decisions of DEIVA: a functionality to both locate as well as identify features in the visualization, emphasis on ease-of-use and ease-of-deployment, permissive free software license, no specific client or server dependencies, and the possibility to extend and integrate it with other systems.

## Availability and requirements

- Project name: DEIVA
- Project home page: https://github.com/Hypercubed/DEIVA
- Archived version: 1.0.0 (https://github.com/Hypercubed/DEIVA/releases/tag/v1.0.0)
- Operating system(s): Platform independent
- Programming language: JavaScript
- License: MIT

## Abbreviations

DEIVA: Differential Expression Interactive Visual Analysis
DGE: Differential gene expression
FOSS: Free and open source software
Hexbin: Hexagonal binning
SPA: Single-page application
